# Effects of Gamification on Assessment of Spatial Release From Masking

**DOI:** 10.1044/2022_AJA-22-00133

**Published:** 2023-02-10

**Authors:** William J. Bologna, Audrey A. Carrillo, David S. Clamage, Laura Coco, Yue J. He, Esteban Sebastian Lelo de Larrea-Mancera, G. Christopher Stecker, Frederick J. Gallun, Aaron R. Seitz

**Affiliations:** a Department of Speech-Language Pathology and Audiology, Towson University, MD; b Brain Game Center, University of California, Riverside; c Oregon Hearing Research Center, Oregon Health and Science University, Portland; d VA Health Services Research & Development (HSR&D) Service Center of Innovation, Center to Improve Veteran Involvement in Care (CIVIC), VA Portland Health Care System, OR; e School of Speech, Language, and Hearing Sciences, San Diego State University, CA; f Department of Psychology, University of California, Riverside; g Center for Hearing Research, Boys Town National Research Hospital, Omaha, NE; h Department of Psychology, Northeastern University, Boston, MA

## Abstract

**Purpose:**

Difficulty understanding speech in noise is a common communication problem. Clinical tests of speech in noise differ considerably from real-world listening and offer patients limited intrinsic motivation to perform well. In order to design a test that captures motivational aspects of real-world communication, this study investigated effects of gamification, or the inclusion of game elements, on a laboratory spatial release from masking test.

**Method:**

Fifty-four younger adults with normal hearing completed a traditional laboratory and a gamified test of spatial release from masking in counterbalanced order. Masker level adapted based on performance, with the traditional test ending after 10 reversals and the gamified test ending when participants solved a visual puzzle. Target-to-masker ratio thresholds (TMRs) with colocated maskers, separated maskers, and estimates of spatial release were calculated after the 10th reversal for both tests and from the last six reversals of the adaptive track from the gamified test.

**Results:**

Thresholds calculated from the 10th reversal indicated no significant differences between the traditional and gamified tests. A learning effect was observed with spatially separated maskers, such that TMRs were better for the second test than the first, regardless of test order. Thresholds calculated from the last six reversals of the gamified test indicated better TMRs in the separated condition compared to the traditional test.

**Conclusions:**

Adding gamified elements to a traditional test of spatial release from masking did not negatively affect test validity or estimates of spatial release. Participants were willing to continue playing the gamified test for an average of 30.2 reversals of the adaptive track. For some listeners, performance in the separated condition continued to improve after the 10th reversal, leading to better TMRs and greater spatial release from masking at the end of the gamified test compared to the traditional test.

**Supplemental Material:**

https://doi.org/10.23641/asha.22028789

Difficulty understanding speech in noise is one of the most common communication problems, affecting nearly all individuals with hearing loss (Noble & Gatehouse, 2006; Souza et al., 2018), as well as older adults with normal hearing thresholds (Beck et al., 2018), and patients with a history of traumatic brain injury (Gallun et al., 2017). Clinical assessment of speech understanding in noise typically involves monaural (one ear) presentation of sentences masked by broadband noise or multitalker babble. Performance on these tests is associated with peripheral hearing sensitivity but is not well predicted by self-reported difficulty in noise (Souza et al., 2018). In order to improve the relationship between clinical assessment and self-reported difficulty, testing methods that more closely resemble the challenges of real-world listening are needed.

One aspect of real-world listening that is absent from many traditional speech-in-noise tests is the use of speech maskers that are spatially separated from target speech. Under these realistic conditions, listeners use spatial cues to separate target speech from other competing talkers, which facilitates speech understanding at unfavorable target-to-masker ratios (TMRs; Bronkhorst, 2015; Bronkhorst & Plomp, 1988; Gallun et al., 2013; Marrone et al., 2008). These spatial cues can be delivered via headphones by manipulating the relative timing and intensity of signals at the two ears (Bronkhorst & Plomp, 1988). Using this approach, speech recognition can be measured with simulated colocated and spatially separated target and maskers, revealing improved speech recognition with spatial cues (spatial release from masking; Gallun et al., 2013; Jakien, Kampel, Stansell, & Gallun, 2017; Marrone et al., 2008). Similar speech recognition tests with spatially oriented maskers have been shown to correlate with self-reported difficulty in noise (Best et al., 2015). Despite the importance of spatial cues to real-world listening, there are few clinical speech-in-noise tests that involve spatialized speech maskers. One exception is the Listening in Spatialized Noise-Sentence Test (LiSN-S; Cameron & Dillon, 2007), which was originally designed as a diagnostic test for auditory processing disorder. While the LiSN-S can assess spatial hearing abilities and has the advantage that the stimuli are natural speech, its open response set format requires the administrator to score patient responses. Considering the time demands of a busy clinic, it would be advantageous to have an automated test of spatial hearing that could be completed while the patient is waiting to be seen by the audiologist. The motivation for this study is to begin developing an automated clinical test of speech recognition with spatially separated speech maskers that will more closely reflect patient-reported difficulty understanding speech in noise.

The significance of signal content to the listener is another important aspect of real-world communication that is not well captured by traditional clinical speech-in-noise tests. Social communication is an ingrained human activity that is intrinsically rewarding, and listeners use considerable effort just to engage in conversations in complex sound environments (Maturana & Varela, 1987; McDermott, 2009). By contrast, even most traditional speech-in-noise tests that use open-set naturalistic speech use sentences with irrelevant content that have limited intrinsic rewards, might not fully engage the listener in the speech recognition task, and might not properly motivate them to perform at the best of their ability. Gibson (1986) offered a similar criticism to picture-based assessment and recommended a study of perception considering its function, that is, adhering to the way the participant uses sensory information to interact with the environment. Under this scheme, the speech stimuli under evaluation need be relevant to the behavior of the participant for the evaluation to be valid and also naturally promote intrinsic reward and motivation. Limited relevance of testing materials, low participant motivation, and unrealistic nonspatialized maskers might all contribute to the poor relationship between traditional speech-in-noise tests and subjective report of communication difficulty. Thus, an important goal for new tests of speech understanding in noise is to include realistic maskers and behaviorally relevant test materials that promote intrinsic sources of motivation and engagement and more consistently capture peak performance of the patient.

Play behavior in games can be intrinsically motivating, can afford highly skilled performance on relatively simple tasks, and can be used in a wide variety of contexts (Green & Seitz, 2015; McGonigal, 2011; Vygotsky, 1933). Thus, different game elements have been applied to nongame laboratory tasks, which are “gamified” with the aim of achieving behaviorally relevant performance (within the context of the game) beyond boredom and anxiety of traditional tasks (see the studies of Csikszentmihalyi, 2000; McGonigal, 2011), and mitigating fatigue, attentional lapses, poorer performance, and variability in scores (Deveau & Seitz, 2014; but see the study of Hawkins et al., 2013).

Gamification refers to the application of game elements developed in the entertainment industry to a context that is not traditionally a game (Deterding, 2012). An example of gamification is embedding perceptual stimuli in a behaviorally relevant interaction within a game metaphor (e.g., you are on a spaceship trying to win a race). The advantages of gamification include increased motivation and engagement of the user, which can address the problems associated with existing tests of speech in noise. Recently, applications of gamification to auditory training have shown improvements in speech reception thresholds in different noise conditions, including speech-on-speech masking in young adults with normal hearing (Lelo de Larrea-Mancera et al., 2021; Whitton et al., 2014) and in older adults with hearing loss (Whitton et al., 2017). These results suggest that introducing game elements into auditory tasks with complex stimuli might achieve the behavioral relevance needed to motivate listeners to fully engage with the sounds they are tested on. While the benefits of gamification might be extended to clinical practice for other auditory tasks, increased distraction to game elements has also been shown to be detrimental to performance (Katz et al., 2014). Thus, gamified elements must be added carefully and iteratively in order to maximize the potential benefits without disrupting the clinical properties of the test.

Following this approach of iterative addition of elements, the work described here focuses on the gamification of an established closed-set automated test of spatial release from masking (SRM). While it does not involve the use of stimuli that are natural and intrinsically motivating, the new test does introduce potentially motivating game elements into the response that the participant makes. To achieve this gamification, a traditional laboratory test of spatialized speech-on-speech masking using the coordinate response measure (CRM; Bolia et al., 2000) was used as the basis of the gamified test. The stimuli and task specifications from the study of Gallun et al. (2013; also Lelo de Larrea-Mancera et al., 2020; Jakien, Kampel, Stansell, & Gallun, 2017; Marrone et al., 2008) were kept the same for one version used in the experiment (referred to as the “traditional SRM”), and visual elements were added to create a gamified version of this test. To achieve a game-like interaction, we combined the SRM test with a relatively simple computer game, “Pipe Mania” (Pipe Mania, 1989). The object of the game (“Pipes Game”) is to correctly respond to speech stimuli in competition with speech maskers to uncover a complete path of pipes from the left side of the board to the right side. The response grid used in the traditional SRM (see Figure 1a) was replaced with a grid of blank tiles (see Figure 1b). When participants made a response based on the acoustic stimuli, the selected tile was replaced with a straight or L-shaped pipe fitting. When a response tile was selected multiple times over the course of a run, the pipe fitting rotated. As participants completed more trials, more pipes were revealed and rotated into position, creating a path from left to right (see Figure 1c). An adaptive algorithm governed the TMR depending on the participant's performance (see the Method section). After 10 reversals of the adaptive track, participants had the option to “solve” the puzzle, which allowed them to select and rotate remaining pieces to complete the pathway without requiring them to respond to acoustic stimuli.

**Figure 1.**
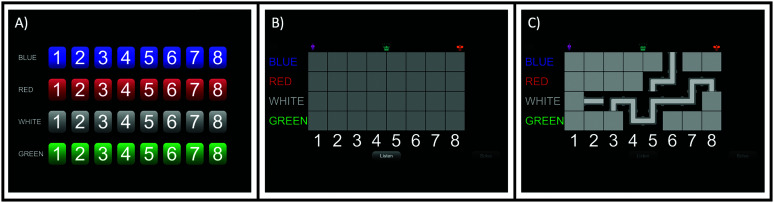
Visual descriptions of the traditional and gamified tests. (A) The response screen for the traditional spatial release from masking test. (B) The game response screen before the first trial has started. (C) The game response screen once several trials have been played and a portion of the pipe path has been revealed. The “Solve” button in the bottom right becomes active after the 10th reversal of the adaptive track and enables participants to complete the path without responding to additional stimuli.

The purpose of this study was to compare spatial release outcomes from the new gamified SRM test, “Pipes Game,” to the traditional laboratory implementation of the test. Young adults with normal hearing completed both the traditional and gamified tests. Importantly, all acoustic and psychometric properties of the test were kept the same in the traditional and gamified implementations, except for the stopping rule (see the Method section). The primary research question was whether estimates of spatial release from masking differed for the traditional and gamified versions of the test. To answer this question, we compared results of the traditional test to the gamified version at the point where the run *would have ended in the traditional test*. We also evaluated how gamified and traditional results differed if thresholds were calculated at the game's natural conclusion (the point at which the participant decided to “solve” the puzzle). The data suggest that listeners were willing to continue playing the game long after the traditional stopping point, leading to better and more consistent thresholds at the end of the game.

## Method

### Participants

There were 64 undergraduate students from the University of California, Riverside, who participated in the study. The participants provided informed consent and received course credit for their participation. All participants self-reported normal hearing. Ten participants were excluded due to incomplete session data, yielding a final sample size of 54 participants (33 female and 22 male participants) with a mean age of 19.62 years old.

### Procedure

The participants were tested remotely with their own equipment (i.e., headphones and mobile device or tablet) and were instructed to complete the testing in a quiet location free of distractions, following the procedures described in the study of Lelo de Larrea-Mancera et al. (2022).^1^ The order of presentation of traditional and gamified SRM was counterbalanced across two groups to ensure that order effects would not bias the comparison between the two tests. In one group (*n* = 25), the participants completed the gamified SRM test in the first session and the traditional SRM test in the second session. In the other group (*n* = 29), the participants completed the tests in the opposite order. The sessions were completed between 1 and 3 days apart. During these sessions, participants completed a demographic questionnaire and the SRM test (either traditional or gamified).

### Measures

Both traditional and gamified SRM tests in colocated and separated conditions were used to measure recognition of target sentences in a multitalker masker. The traditional SRM test showed a grid of four colors by eight numbers for participants to identify and select the positions indicated by target sentences (see Figure 1a). The gamified SRM test presented a grid of blank tiles with four colors on the left and eight numbers in the bottom (see Figure 1b); participants identified and revealed the pathways under the tiles indicated by target sentences to connect pipes from the left side of the screen to the right side (see Figure 1c). Target sentences spoken by a male talker all started with a call sign “Ready Charlie” and two keywords, a color and a number, at a fixed nominal level of 65 dB.^2^ Simultaneously with the target, two maskers spoken by male talkers stated two masking sentences with different call signs and keywords. The target and masker talkers were selected randomly from trial to trial from a set of three male talkers. In the colocated condition, a target talker and two maskers were all perceptually located directly in front of the listeners. In separated condition, head-impulse responses were convolved around the two maskers to simulate the perception of spatial locations at 45° to the left and to the right of the target talker, as described in the study of Gallun et al. (2013). For both traditional and gamified SRM tests, the target level was fixed at a nominal level of 65 dB. The masker levels were adaptive with a one-up/one-down procedure on a linear scale in steps of 2 dB, and each started at an initial value of 10 dB TMR (i.e., 10 dB below the level of the target).

The traditional and gamified SRM tests had different stopping rules. The traditional test stopped after 10 reversals, whereas the gamified test continued until participants had at least 10 reversals and completed the puzzle. Participants could complete the puzzle by responding to more trials to create a complete path from left to right or by pressing the “solve” button that appeared after 10 reversals. In rare cases (2.8% of runs), a participant solved the puzzle before getting 10 reversals, in which case a new grid of blank tiles was generated, and the adaptive track continued until they solved the new puzzle. In the traditional SRM test, a threshold (TMR) is calculated from the average of the last six reversals. In the gamified version, thresholds were calculated in two ways: first using reversals 4–10, which facilitated a direct comparison with thresholds from the traditional test, and then again using the final six reversals to explore how performance changed over the course of longer runs in the gamified test.

## Results

This study evaluated the effects of adding gamified elements to the traditional SRM test. All participants completed both the traditional SRM test and the gamified version in a counterbalanced order. In each version of the test, participants completed one adaptive track in the colocated condition and one adaptive track in the separated condition. The traditional adaptive track ends after 10 reversals, whereas the gamified version does not end until the participant solves the puzzle, potentially leading to tracks with more than 10 reversals.

The gamified runs took an average of 4.5 min to complete (*SD* = 2.2 min) and had an average of 30.2 reversals (*SD* = 16.2). For comparison, the traditional runs took an average of 1.5 min to complete (*SD* = 0.3 min) and always contained 10 reversals. To facilitate comparisons with the traditional test, gamified thresholds were calculated based on the average of reversals 4–10 (as in the traditional test). For each participant, spatial release was calculated as the difference between TMR for the colocated and separated conditions from the traditional and gamified thresholds.

**Figure 2.**
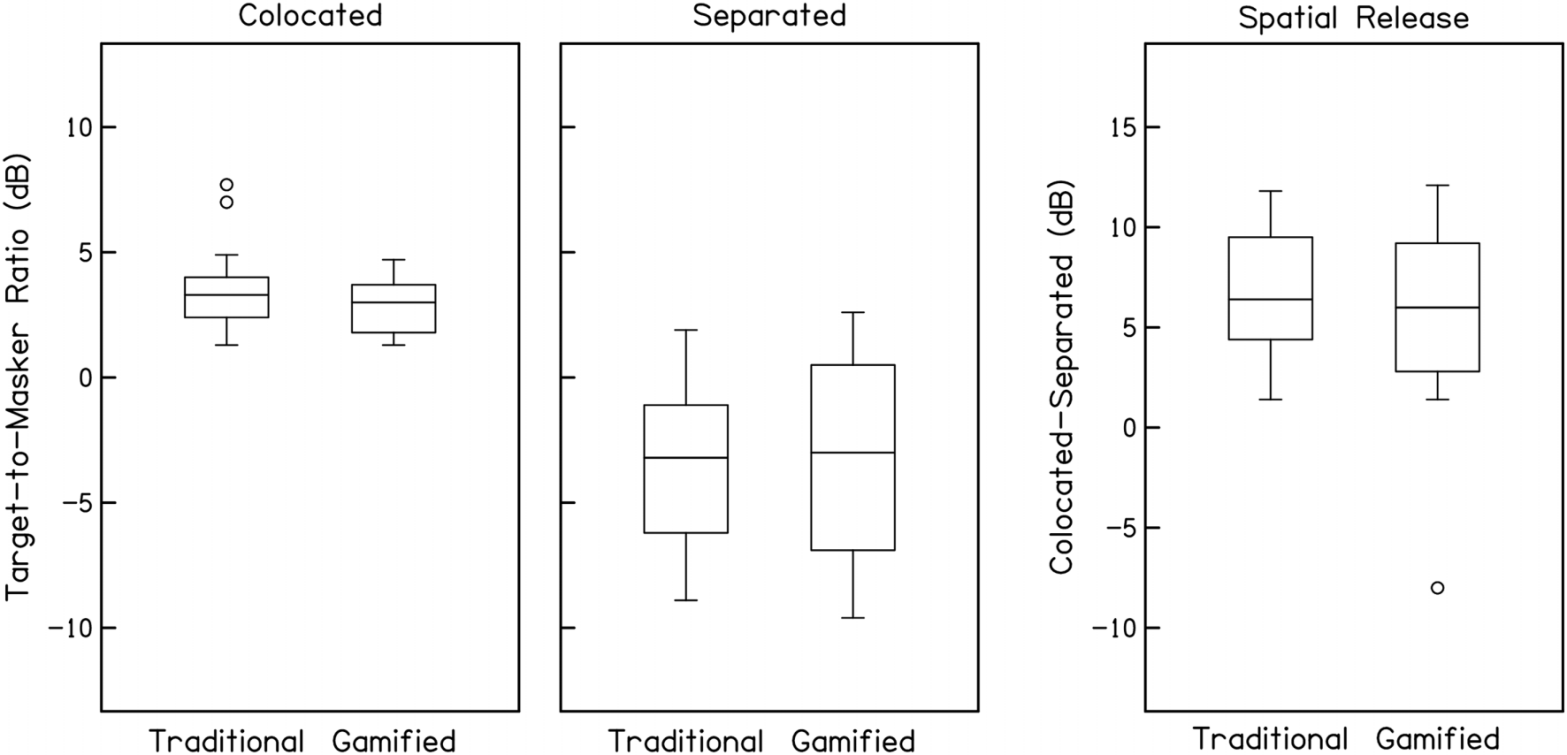
Target-to-masker ratio thresholds (dB) for the traditional and gamified tests with colocated maskers (left panel), spatially separated maskers (middle panel), and spatial release from masking (colocated–separated; right panel). Data are collapsed across test order. For each distribution, the middle line indicates the median, edges of boxes indicate 1st and 3rd quartiles, whiskers indicate 10th and 90th percentiles, and circles indicate outliers (more than 1.5 × interquartile range above or below quartiles). Similar performance and estimates of spatial release were observed for traditional and gamified tests.

The primary goal of the study was to determine if the gamified test produced similar estimates of spatial release as the traditional test. The distribution of TMRs for the traditional and gamified versions of the test in the colocated and separated conditions are displayed in the two left panels in Figure 2, with spatial release from masking (colocated–separated) in the right panel. A paired-samples *t* test comparing spatial release for the traditional and gamified tests was nonsignificant, *t*(53) = 0.75, indicating that the two tests produced similar estimates of spatial release.

**Figure 3.**
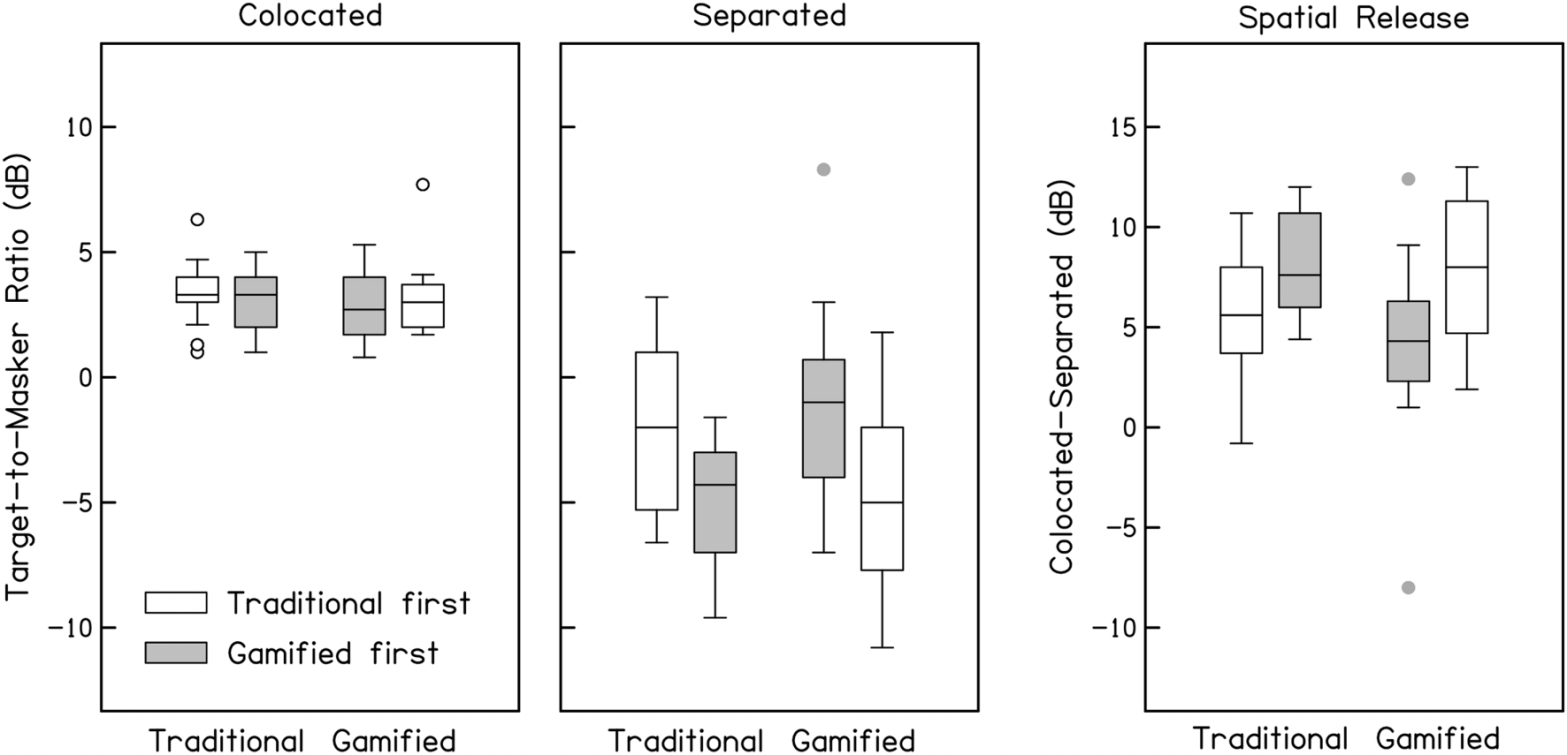
Target-to-masker ratio thresholds (dB) for the traditional and gamified tests with colocated maskers (left panel), spatially separated maskers (middle panel), and spatial release from masking (colocated–separated; right panel), with test order as a parameter (traditional first in white, gamified first in gray). For each distribution, the middle line indicates the median, edges of boxes indicate 1st and 3rd quartiles, whiskers indicate 10th and 90th percentiles, and circles indicate outliers (more than 1.5 × interquartile range above or below quartiles). Performance with separated maskers and estimates of spatial release improved for the second test, regardless of test order.

Previous investigations of the SRM test have demonstrated that TMRs and spatial release typically improve over several repeated runs (Lelo de Larrea-Mancera et al., 2020; Jakien, Kampel, Stansell, & Gallun, 2017). This learning effect is also apparent in these data as an effect of test order: Participants who completed the traditional test first had better spatial release on the gamified test and vice versa. To examine whether gamification influences the pattern of task learning, data from Figure 2 are replotted in Figure 3 separately for participants who completed the traditional test first (unfilled boxes) and the gamified test first (gray-filled boxes). Average thresholds and spatial release are shown in Table 1. These data were analyzed with three separate two-way analysis of variance (ANOVAs^3^; colocated, separated, and spatial release), each with factors of run (first run vs. second run) and test (traditional vs. gamified). The significance threshold for these tests was set conservatively at .0167 to account for the three parallel comparisons. For the colocated condition, neither factor nor their interaction was significant, run: *f*(1) = 0.34; test: *f*(1) = 0.75; Run × Test: *f*(1) = 1.02. In the separated condition, there was evidence of a practice effect; the run factor was significant, *f*(1) = 15.80, *p <* .001, but test factor and the interaction between test and run were not significant, test: *f*(1) = 0.40; Run × Test: *f*(1) = 0.01. Spatial release results mirrored the separated condition, with a main effect of run, *f*(1) = 14.22, *p <* .001, and no effect of test or the interaction, test: *f*(1) = 0.91; Run × Test: *f*(1) = 0.24. This pattern of results suggests that listeners performed better on the second run of the separated condition than the first, regardless of the order of the two tests (traditional or gamified first). In contrast, performance in the colocated condition was stable across two runs. In general, these results indicate that the traditional and gamified tests provide similar TMR data, and both tests are characterized by a learning effect wherein performance in the separated condition improves from the first run to the second.

**Table 1.** Average and standard deviation of thresholds and spatial release across conditions for individual runs of the traditional and gamified tests.

Variable			Threshold		Spatial release	
Condition	Test	Run	*M*	*SD*	*M*	*SD*
Colocated	Traditional	First	3.5	1.2		
Colocated	Traditional	Second	3.0	1.6		
Colocated	Gamified	First	2.9	1.8		
Colocated	Gamified	Second	3.1	1.4		
Colocated	Gamified (Last 6)	First	3.5	2.6		
Colocated	Gamified (Last 6)	Second	3.2	1.6		
Separated	Traditional	First	−2.0	3.9	5.5	4.0
Separated	Traditional	Second	−5.1	3.3	8.1	3.5
Separated	Gamified	First	−1.5	4.2	4.4	4.0
Separated	Gamified	Second	−4.7	4.6	7.7	4.5
Separated	Gamified (Last 6)	First	−5.4	4.3	8.9	5.1
Separated	Gamified (Last 6)	Second	−6.0	4.4	9.2	4.4

*Note.* Spatial release is calculated as the difference between the colocated and separated thresholds for an individual participant on the traditional or gamified test. Run indicates whether participants completed the gamified test first and the traditional test second (*N* = 25) or completed the tests in the opposite order (*N* = 29).

### Gamified Thresholds Estimated From Last Six Reversals

**Figure 4.**
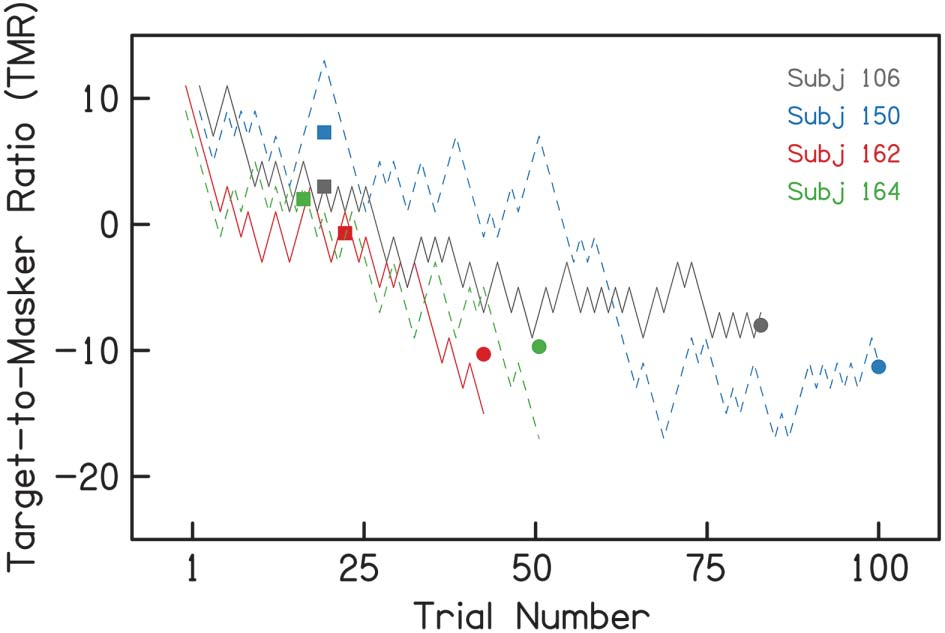
Example adaptive tracks from four participants in the separated condition of the gamified test. Target-to-masker ratio (TMR) on each trial is plotted as a function of trial number. Lines are offset vertically (by 1 dB TMR) and horizontally (by 1 trial) for clarity. Squares indicate TMR thresholds calculated from the point of the 10th reversal, and circles indicate TMR thresholds calculated from the last six reversals. In each example, performance improved over the course of the run beyond the 10th reversal.

**Figure 5.**
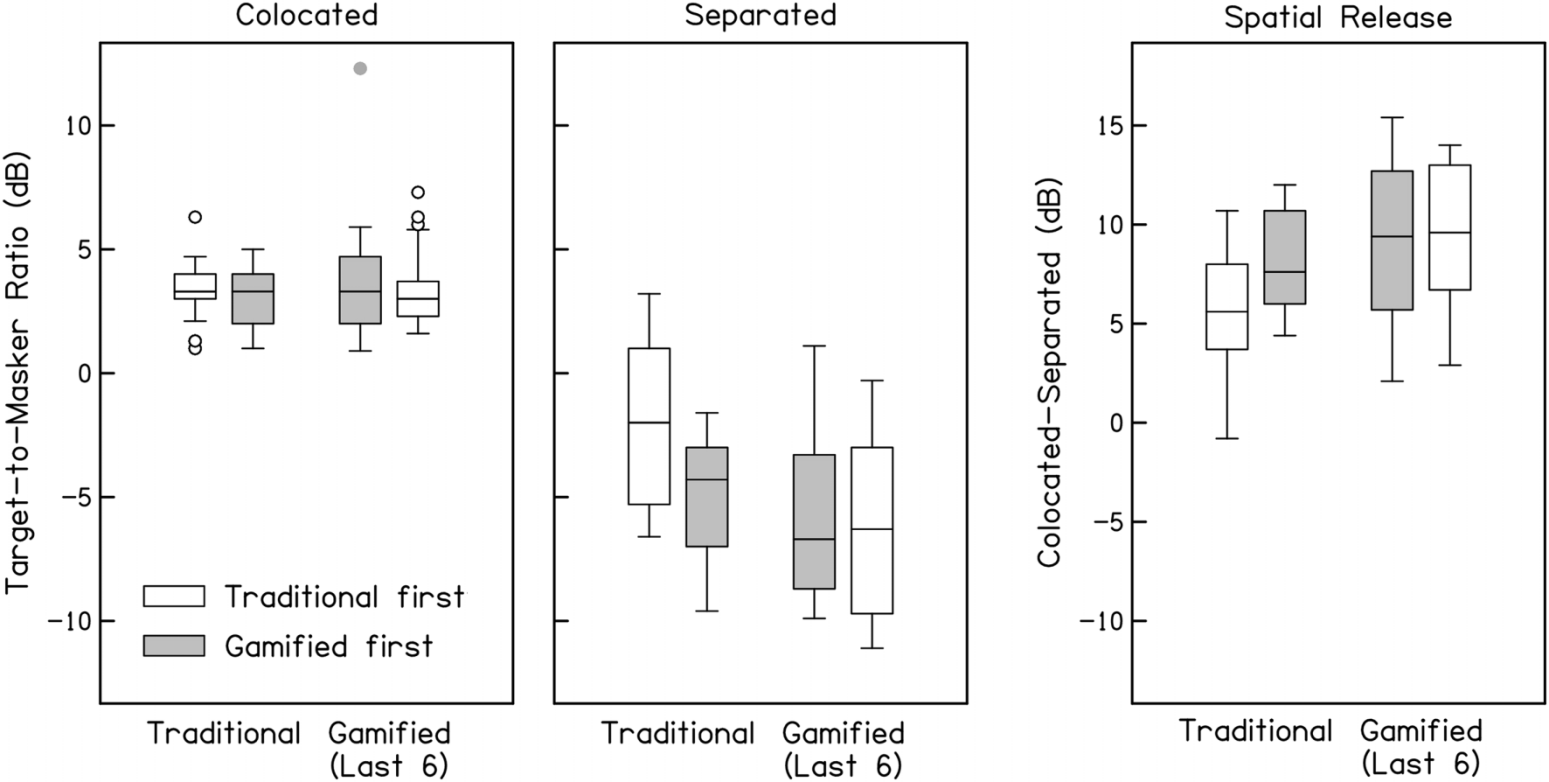
Target-to-masker ratio thresholds (dB) calculated from the last six reversals in the traditional and gamified tests with colocated maskers (left panel), spatially separated maskers (middle panel), and spatial release from masking (colocated–separated; right panel), with test order as a parameter (traditional first in white, gamified first in gray). For each distribution, the middle line indicates the median, edges of boxes indicate 1st and 3rd quartiles, whiskers indicate 10th and 90th percentiles, and circles indicate outliers (more than 1.5 × interquartile range above or below quartiles). Performance in the separated condition and estimates of spatial release were better for the gamified test than those for the traditional test. Improvements associated with test order are reduced when thresholds are calculated from the last six reversals, particularly for participants who completed the gamified test first (gray bars).

As aforementioned, the traditional test ended after the 10th reversal, whereas the gamified test continued until the participant elected to solve the puzzle, which lasted 30.2 reversals on average. Visual inspection of the adaptive tracks from the gamified runs indicated that performance continued to improve after the 10th reversal for many participants (examples are shown in Figure 4). To determine whether these additional trials yielded better performance at the group level, thresholds were recalculated from the final six reversals of the adaptive tracks for the gamified runs, referred to below as “Last 6” thresholds. These data are presented in Figure 5 alongside data from the traditional test (same as Figure 3), and average thresholds and spatial release are shown in Table 1. Similar to the results reported above, these data were analyzed with three separate two-way ANOVAs^4^ (colocated, separated, and spatial release), each with factors of run (first run vs. second run) and test (traditional vs. gamified), and a significance threshold of .0167. The results in the colocated condition were similar to those described above; neither factor nor their interaction was significant, run: *f*(1) = 1.10; test: *f*(1) = 0.08; Run × Test: *f*(1) = 0.04. In the separated condition, there was a significant effect of run, *f*(1) = 6.75, *p <* .0167, and test, *f*(1) = 7.83, *p <* .0167, but no significant interaction, Run × Test: *f*(1) = 2.38. Spatial release results indicated a main effect of test, *f*(1) = 7.48*, p < .*01, but no effect of run and no interaction, run: *f*(1) = 3.96; Run × Test: *f*(1) = 0.18. In contrast to the results described previously, “Last 6” thresholds indicated that better performance in the separated condition and greater spatial release was measured with the gamified test than the traditional test. While the effect of run remained significant in the separated condition, it no longer reached significance in the measure of spatial release from masking.

## Discussion

Traditional speech-in-noise tests are not representative of real-world listening, and their results often fail to correlate with subjective reports of communication difficulty in noise. One factor that may contribute to the poor relationship between test results and subjective report is that listeners are not engaged and motivated during a traditional speech-in-noise test to the same extent that they are in everyday communication. Gamification might be a means of increasing participant engagement with the task and stimulus, attaining motivation to perform during testing, thereby improving the relationship between test results and patient's real-world experiences. The goal of this study was to evaluate the effects of adding simple gamified elements to a traditional measure of spatial release from masking.

One initial concern with gamification was the possibility that adding gamified elements would disrupt the validity of the task. In some applications, gamification can distract from and decrease task performance (Katz et al., 2014). In this study, gamified elements were limited to visual effects (replacing traditional response grid with array of pipe tiles) and a puzzle-based goal (connect pipes from the left side of the screen to the right side). We considered the possibility that adding a visual element to the SRM test might provide a visual cue that could aid performance. Toward the end of a run, participants might have been able to guess more effectively by recognizing the tile pattern and predicting which spaces would need to be selected next. Results from our current study indicate that these simple gamified elements did not affect TMRs or estimates of spatial release if thresholds were calculated from the same point on the adaptive track as the traditional test. While the gamified TMRs estimated from the last six reversals were better than traditional TMRs, this effect is unlikely to be due to a visual cue from the game board. If participants were using a visual cue to improve their performance at the end of the run, we would expect better TMRs in the gamified test than the traditional test for both colocated and separated conditions, as both conditions would provide this visual cue. These data indicated an improvement in last six thresholds only in the separated condition; colocated TMRs were virtually identical for the traditional test and the gamified test (calculated at either the 10th or last six reversals). Thus, it seems more likely that the improvement in last six thresholds in the separated condition was due to participants learning to use the spatial cue, rather than benefiting from the visual cue once the pipes pattern is mostly revealed. Nevertheless, future iterations of the gamified test of spatial release will provide a points-based reward for solving the puzzle earlier in the run to avoid a potential visual cue.

Based on these initial results future work will include additional gamified elements, such as a more compelling game metaphor, and a point system that rewards correct responses. One common gamification element is additional rewards for “streaks” of correct responses. In the context of an adaptive psychometric task, streaks are important for efficiently reaching peak performance, especially if run length is determined by a specific number of reversals. This concept is visible in Figure 4, where some runs are characterized by a streak of correct responses occurring *after the 10th reversal*. These streaks put the participant into the range of TMRs that more accurately reflects their peak performance on the task than their threshold at the 10th reversal. Considering the importance of these streaks, future iterations will include point-based rewards for streaking to encourage this behavior through gamification. By iteratively adding gamified elements, we can determine how to best facilitate task performance and improve the relationship between test results and subjective reports of difficulty in noise.

At the group level, traditional and gamified TMRs were similar at the point of the 10th reversal, but participants performed better in the separated condition on the second testing session than the first. Similar learning effects for the traditional SRM test have been reported previously (Lelo de Larrea-Mancera et al., 2020; Jakien, Kampel, Stansell, & Gallun, 2017), hence, the order of tests was counterbalanced to ensure that this learning effect would not influence the comparison between traditional and gamified tests. In this study, the learning effect persisted for multiple days (test sessions occurred 1–3 days apart), and learning transferred from traditional to gamified tests and vice versa. These results suggest that young listeners with normal hearing benefit from extended exposure to the spatially separated stimuli and may learn to use the spatial cue and maximize their performance throughout testing in the separated condition. This learning effect is apparent after only one run of 10 reversals using the traditional test but can also be seen over the course of a single longer run of the gamified test.

Thresholds calculated from the end of the gamified tracks indicated better thresholds in the separated condition, greater spatial release from masking, and a reduced learning effect. One explanation for this pattern of results is that the longer gamified runs allowed participants time to learn how to use the spatial cue to improve their TMR in the separated condition. Gamified runs were roughly three times longer than traditional runs (4.5 min with 30.2 reversals, compared to 1.5 min with 10 reversals). While increased test time is not desirable from a clinical perspective, a single gamified run captured a level of performance in the separated condition that could only be achieved after multiple runs with 10 reversals. Thus, the gamified TMRs measured from the last six reversals better reflect listeners' maximum performance and resulted in more consistent estimates of spatial release than thresholds estimated from 10 reversals. This interpretation is consistent with the pattern observed in the adaptive tracks of the gamified runs (see Figure 4).

It is unclear from these data whether increased task engagement through gamification affected task learning or simply facilitated testing over a greater number of trials. The pattern of results in the adaptive tracks suggested that some listeners learned to use the spatial cue more slowly than others over the course of a run (see Figure 4). Considering the differences in run length, the improvements in separated TMRs over the course of the gamified run might be related to increased exposure to the stimuli, rather than gamification, per se. The current results indicate that participants continue to improve their performance in the separated condition beyond the 10th reversal, and that the additional trials afforded by the gamified test allowed these listeners to maximize their performance. Gamification should support task learning to the extent that it facilitates engagement of participants in the test over more trials. In Pipes Game, participants could “solve” the puzzle at any time after the 10th reversal, which would effectively end the adaptive track. Interestingly, most participants elected to continue listening after the solve option became available, which suggests they were enjoying the game or became fixated on using the auditory signals to complete the puzzle. We did not allow the traditional task to be performed for an extended number of trials so we cannot test whether the gamified task promoted acceptance and willingness to continue engaging with test materials. Previous studies have shown increased acceptance of gamified experimental tasks even in the absence of performance differences with traditional nongamified tasks (Hawkins et al., 2013). Future work will investigate whether gamification increases the number of trials that listeners are willing to perform relative to a nongamified test. These data will help determine whether gamification accelerates task learning of the use of spatial cues, facilitates testing over more trials, or both.

An important next step toward increasing realism and participant engagement in testing is to improve the content of the speech material. The CRM corpus uses synthetic sentences that are not characteristic of real-world communication. This corpus was selected for this initial investigation because the closed response set allows for automated scoring, and results could be compared to previous research on spatial release using CRM sentences (i.e., Jakien, Kampel, Gordon, Gallun, 2017; Jakien, Kampel, Stansell, Gallun, 2017). However, the structure of the CRM sentences imposes several unrealistic constraints on the speech-in-noise task, such as synchronized keywords, the absence of contextual cues, and target identification based on an auditory callsign. Work is currently underway to address these limitations with gamified tests that use more realistic speech material, such as Bamford–Kowal–Bench sentences (Bench et al., 1979). Use of more natural sentences and competing signals might improve the relationship between test results and subjective reports of difficulty (Best et al., 2015), which will support our goals with gamification.

The participants in this study were young adults with normal hearing, and this population is less likely to experience communication difficulty in noise than most patients completing clinical speech-in-noise tests. An important step forward in this work is to begin testing populations where speech-in-noise difficulty is common, such as older adults (Humes, 2021), individuals with hearing loss (Noble & Gatehouse, 2006; Souza et al., 2018), and individuals with history of traumatic brain injury (Gallun et al., 2017). In these populations, the correlation between gamified test results and subjective reports of difficulty has important clinical relevance. Future work will evaluate gamification-based tasks among diverse patient populations with the goal of accurately approximating patients' subjective reports of listening difficulty.

## Conclusions

In this initial investigation of the effects of gamification on assessment of spatial release from masking, gamified elements were conservatively added to the traditional SRM test. Results indicated that the gamified version produced similar TMRs and estimates of spatial release compared to the traditional test when thresholds were calculated from the same point on the adaptive track. Gamified runs contained additional trials after the 10th reversal, and analyses of thresholds calculated from the last six reversals revealed improved performance with spatially separated maskers and greater spatial release from masking than thresholds estimated from the point of the 10th reversal. With the current data set, these potential effects of gamification on task learning cannot be disentangled from exposure effects from testing over a greater number of trials. Future development and research on this gamified test of spatial release will include new gamified features and assessment of different populations to determine whether gamification improves the relationship between test results and subjective reports of difficulty in noise.

## Author Contributions

**William J. Bologna:** Data curation (Lead), Formal analysis (Lead), Writing – original draft (Lead), Writing – review & editing (Equal). **Audrey A. Carrillo:** Data curation (Equal), Methodology (Equal), Project administration (Lead), Writing – review & editing (Equal). **David S. Clamage:** Software (Lead). **Laura Coco:** Writing – review & editing (Equal). **Yue J. He:** Data curation (Supporting), Methodology (Equal), Writing – review & editing (Equal). **Esteban Sebastian Lelo de Larrea-Mancera:** Writing – review & editing (Equal). **G. Christopher Stecker:** Conceptualization (Equal), Funding acquisition (Equal), Writing – review & editing (Equal). **Frederick J. Gallun:** Conceptualization (Equal), Funding acquisition (Equal), Supervision (Equal), Writing – review & editing (Equal). **Aaron R. Seitz:** Conceptualization (Equal), Funding acquisition (Equal), Supervision (Equal), Writing – review & editing (Equal).

## Data Availability Statement

Deidentified data are available from the corresponding author on request. Traditional and gamified tests of spatial release from masking are also available for download on request.

## Supplementary Material

10.1044/2022_AJA-22-00133SMS1 Supplemental Material S1 Describes a parallel set of analyses of TMR data (10th reversal and last 6 thresholds) using linear mixed effects modeling, including the modeling approach, model testing, and interpretation of the results. Modeling results are consistent with the ANOVAs reported in the main text.

## Footnotes

1 Participants interacted with the study team via a videoconferencing application on their computer and downloaded the free test application (BGC Science) on their own phone or tablet. Headphones were required to be used and verified by the study team via the video image, but the type of headphone was not controlled. This method is supported by the results in the study of Lelo de Larrea-Mancera et al. (2022), who found no significant influence of equipment or test environment on the traditional SRM tested in this manner.

2 As the systems used were not calibrated, only the relative levels of the targets and maskers can be specified with certainty. Thus, the levels reported are referred to as nominal dB. However, Lelo de Larrea-Mancera et al. (2022) found that performance was not statistically different for listeners using calibrated and uncalibrated systems. Whereas overall intensity can influence spatial release below 20-dB sensation level, due to reduced audibility of high-frequency cues (Jakien, Kampel, Gordon, & Gallun, 2017; Rana & Buchholz, 2018), at the suprathreshold levels used here, spatial release is consistent even when headphones reduce overall level by more than 10 dB (Lelo de Larrea-Mancera et al., 2020).

3 TMR data were also analyzed using a linear mixed-effects model with factors of condition (colocated vs. separated), run (first run vs. second run), and test (traditional vs. gamified), with subject as a random effect term. The modeling approach, model testing, and interpretation are described in Supplemental Material S1. Modeling results were consistent with the patterns observed in the ANOVA analyses.

4 Last six TMR data were also analyzed using a linear mixed-effects model with factors of condition (colocated vs. separated), run (first run vs. second run), and test (traditional vs. gamified), with subject as a random effect term. The modeling approach, model testing, and interpretation are described in Supplemental Material S1. Modeling results were consistent with the patterns observed in the ANOVA analyses.
