# Supplementary material for: Effects of Gamification on Assessment of Spatial Release From Masking
Source: Am J Audiol. 2023 Feb 10;32(1):210–9. doi: 10.1044/2022_AJA-22-00133 (PMC10171850; doi:10.1044/2022_AJA-22-00133)
Supplement: Supplemental Material S1 [file AJA-32-210-s001.pdf]

Supplemental Material S1. Describes a parallel set of analyses of TMR data (10th reversal and last 6 thresholds) using linear mixed effects modeling, including the modeling approach, model testing, and interpretation of the results. Modeling results are consistent with the ANOVAs reported in the main text.

Statistical Analysis With Linear Mixed Effects Modeling

A parallel set of analyses was conducted with a logistic regression of TMR data implemented in R (R Core Team, 2021) using linear mixed effects models (LMM; R-package: lme4; Bates et al., 2015). Separate LMMs were completed for TMR thresholds calculated from the point of the 10th reversal and for Last 6 thresholds. The LMMs specified TMR threshold of an individual as the dependent variable and estimated separate β coefficients for each independent variable included in the model. The same modeling approach was used to generate the final two models. First, an omnibus model was generated with all the following design-level factors and interactions included: Condition (0 for colocated, 1 for separated), Run (0 for first run, 1 for second run), Test (0 for traditional, 1 for gamified), all two- and three-way interactions between these factors, and a random subject effects term (Subj). A simplified version of the omnibus LMM is expressed as:

$$TMR \sim Condition \times Run \times Test + (Subj)$$

The second step in our modeling approach was a stepwise process of factor elimination using model testing (Hofmann, 1997) to optimize the model to retain only the interactions that significantly improve model fit. The third step was to test the significance of fixed effects using model testing and restructure the significant interactions such that the final model best described the data with the fewest number of factors. The final models mirror the pattern of results in the ANOVAs reported in the main text.

10th Reversal TMRs

Results of the omnibus model for the 10th reversal thresholds are displayed in Table 1. Model testing confirmed a significant contribution of the Condition × Run interaction ($\chi^2 = 16.31$, $p < .001$), but other two- and three-way interactions did not significantly improve the fit of the model (all $\chi^2 < 1.07$, nonsignificant in all cases). Subsequent model testing confirmed significant contributions of Condition ($\chi^2 = 169.40$, $p < .001$) and Run ($\chi^2 = 17.74$, $p < .001$), but not Test ($\chi^2 = 0.11$, nonsignificant). As such, Test was removed from the final model, and the effect of Run was split into the two halves of its interaction with Condition. A simplified version of the final LMM is expressed as:

$$TMR\_10 \sim Condition + (Condition \times Run) + (Subj)$$

Results of the final model for 10th reversal TMRs are displayed in Table 2. Modeling results indicated that TMRs were better in the separated condition than the colocated condition ($\beta_{\text{Condition}} = –4.98$; $t = –9.71$). In the separated condition, TMRs were better for the second run than the first run ($\beta_{\text{Separated×Run}} = –3.09$; $t = –6.02$), whereas TMRs were essentially equivalent for first and second runs in the colocated condition ($\beta_{\text{Colocated×Run}} = –0.17$; $t = –0.33$). These results are consistent with pattern of data in Figure 3 and the interpretations of ANOVA results reported in the main text.

Last 6 TMRs

Results of the omnibus model for Last 6 thresholds are displayed in Table 3. Model testing confirmed a significant contribution of the Condition × Run interaction ($\chi^2 = 12.12$, $p < .01$), and the Condition × Test interaction ($\chi^2 = 15.90$, $p < .001$), but other two- and three-way interactions did not significantly improve the fit of the model (both $\chi^2 < 2.26$, nonsignificant in both cases). Subsequent model testing confirmed significant contributions of Condition ($\chi^2 = 212.21$, $p < .001$), Run ($\chi^2 = 8.29$, $p < .01$), and Test ($\chi^2 = 6.84$, $p < .01$). Considering the significant two-way interactions, the effects of Run and Test were split into the two halves of their interactions with Condition. A simplified version of the final LMM is expressed as:

TMR_Last6 ~ Condition + (Condition × Run) + (Condition × Test) + (Subj)

Results of the final model for 10th reversal TMRs are displayed in Table 4. Modeling results indicated that TMRs were better in the separated condition than the colocated condition ($\beta_{\text{Condition}} = -6.02$; $t = -9.39$). In the separated condition, TMRs were better for the second run than the first run ($\beta_{\text{Separated×Run}} = -1.84$; $t = -3.42$), whereas TMRs were essentially equivalent for first and second runs in the colocated condition ($\beta_{\text{Colocated×Run}} = -0.37$; $t = -0.69$). Similarly, separated TMRs were better in the gamified test than the traditional test ($\beta_{\text{Separated×Test}} = -2.16$; $t = -4.02$), whereas colocated TMRs were essentially equivalent for the two tests ($\beta_{\text{Colocated×Test}} = 0.10$; $t = 0.19$). These results are consistent with pattern of data in Figure 4 and the interpretations of ANOVA results reported in the main text.

Table S1. Omnibus LMM of 10th reversal TMRs with standard estimates, standard error, and *t* values for each fixed effect and interaction term.

Factor	Coding Scheme	Standard Estimate (*β*)	Standard Error	*t* Value
(Intercept)	NA	3.47	0.57	6.14
Condition	0 = colocated; 1 = separated	–5.50	0.70	–7.82
Test	0 = Traditional; 1 = Gamified	–0.54	0.83	–0.65
Run	0 = First Run; 1 = Second Run	–0.44	0.83	–0.53
Condition × Test	1 = Separated & Gamified	1.12	1.03	1.09
Condition × Run	1 = Separated & Second Run	–2.59	1.03	–2.51
Test × Run	1 = Gamified & Second Run	0.58	1.30	0.45
Condition × Test × Run	1 = Separated & Gamified & Second Run	–0.77	1.46	–0.52

Table S2. Final LMM of 10th reversal TMRs with standard estimates, standard error, and *t* values for each fixed effect and interaction term.

Factor	Coding Scheme	Standard Estimate (*β*)	Standard Error	*t* Value
(Intercept)	NA	3.22	0.41	7.82
Condition	0 = colocated; 1 = separated	–4.98	0.51	–9.71
Separated × Run	1 = Separated & Second Run	–3.09	0.51	–6.02
Colocated × Run	1 = Colocated & Second Run	–0.17	0.51	–0.33

Table S3. Omnibus LMM of Last 6 reversal TMRs with standard estimates, standard error, and *t* values for each fixed effect and interaction term.

Factor	Coding Scheme	Standard Estimate (β)	Standard Error	*t* Value
(Intercept)	NA	3.47	0.58	6.03
Condition	0 = colocated; 1 = separated	–5.50	0.73	–7.55
Test	0 = Traditional; 1 = Gamified	0.03	0.85	0.03
Run	0 = First Run; 1 = Second Run	–0.44	0.85	–0.52
Condition × Test	1 = Separated & Gamified	–3.38	1.07	–3.16
Condition × Run	1 = Separated & Second Run	–2.59	1.07	–2.42
Test × Run	1 = Gamified & Second Run	0.14	1.31	0.11
Condition × Test × Run	1 = Separated & Gamified & Second Run	2.24	1.52	1.48

Table S4. Final LMM of Last 6 TMRs with standard estimates, standard error, and *t* values for each fixed effect and interaction term.

Factor	Coding Scheme	Standard Estimate (β)	Standard Error	*t* Value
(Intercept)	NA	3.44	0.49	6.99
Condition	0 = colocated; 1 = separated	–6.02	0.64	–9.39
Separated × Run	1 = Separated & Second Run	–1.84	0.54	–3.42
Colocated × Run	1 = Colocated & Second Run	–0.37	0.54	–0.69
Separated × Test	1 = Separated & Gamified	–2.16	0.54	–4.02
Colocated × Test	1 = Colocated & Gamified	0.10	0.54	0.19

References

Bates, D., Mächler, M., Bolker, B., & Walker, S. (2015). Fitting linear mixed-effects models using lme4. *Journal of Statistical Software*, 67(1), 1–48. https://doi.org/10.18637/jss.v067.i01

Hofmann, D. A. (1997). An overview of the logic and rationale of hierarchical linear models. *Journal of Management*, 23(6), 723–744. https://doi.org/10.1016/S0149-2063(97)90026-X

R Core Team. (2021). *R: A language and environment for statistical computing*. R Foundation for Statistical Computing, Vienna, Austria. https://www.R-project.org/
